# Dr. Stanley Jenkins

**Published:** 1913-06

**Authors:** 


### Obituary.

### DR. STANLEY JENKINS.

It is with very deep regret that news has been received of the sad death of Dr. H. Stanley Jenkins, one of the most distinguished students of the Bristol Medical School. He was the son of Mr. F. A. Jenkins and the great-nephew of the late Dr. Robert Fletcher, of the Surgeon-General's Library, Washington. After working at the Bristol General Hospital, Dr. Jenkins took the Conjoint Qualification in 1898, proceeding in 1903 to the M.D. London and the F.R.C.S. Since 1904 he had devoted himself to the life of a medical missionary in China, where in April of this year he succumbed to an attack of typhus fever. From Dr. Thomas Scollay, a colleague of Dr. Jenkins's at the English Baptist Mission, Sianfu, Shensi, North China, we have received the following short account of Dr. Jenkins's work and last illness, which we are glad to be able to publish :—

"On the 6th of April Dr. H. Stanley Jenkins, of the Baptist Missionary Society, passed away in his thirty-ninth year at the Mission Hospital, Sianfu, Shensi, China. He had just shortly returned to the hospital which he had been in charge of for nearly nine years.

"He first arrived in the year 1904, when China was just settling down after the Boxer rising, and the natives were beginning to realise that the Foreign Missionary only desired to help them. The Chinese among whom he worked will miss him much, for they recognised his skill in treating them when they needed his help, and at every time found in him a true friend whom they could consult on matters not medical. The

Society to which he belonged will miss him much, for while men of his ability are not too numerous, there are few who are so willing to give their years and talents to practising in the heart of China.

"In 1904 there were few railways in China, and the inland journey to Sianfu then took a good month. Immediately on his arrival Dr. Jenkins found his hands full of work, but with a willing heart he took up his task. The difficult language, the despair of so many, proved easy to him, and he soon won the hearts of the people. There is nothing that pleases a Chinese so well as finding someone who is willing to listen to his story. Dr. Jenkins proved a patient listener, and it did one good on out-patient days to see him never showing a trace of weariness as a long story was poured into his ears, most of it irrelevant to the case in question.

"Medical work in Sianfu is very different to what one is accustomed to in England. To begin with, the cases one sees are different. Of course, the Chinese have the same diseases as the English, with some few additions, but those same diseases are seen very often in more advanced stages. Wounds are seen full of maggots, and malignant disease rarely seen until long past the operable stage. The reason for this is that the foreign doctor is looked upon as the last hope. Very often every means of native help is first sought, and only the most serious cases fall into the missionaries' hands. A man will come with a plaster covering his shoulder. This is the native treatment for dislocated shoulder-joint, and has been tried for perhaps six months before the Mission doctor sees it. Besides the plaster the native doctor has one other method of treating such surgical cases. They insert needles to let out the evil humours. Thus it was in surgery that Dr. Jenkins first found scope for his energies.

"The native doctor is not without some knowledge of drugs and their uses. His medicine chest contains many common vegetable aperients, digitalis and many others in the B.P., but all in very crude form. An infusion of the herb in a pint of water would generally be the dose. This proved a great difficulty when using foreign concentrated drugs. The handy little tablet proved rather a drawback.

"There were other and greater difficulties, however. The pioneer doctor finds that he must be surgeon, house-surgeon, nurse and ward servant until he has trained his own assistants. He must also dispense his own medicines, but fortunately the Chinese take well to dispensing. Antiseptic methods, however, are by no means easy for them to learn, and so naturally cases go septic much more readily than at home. Dr. Jenkins, therefore, found his operations limited until he had trained the Chinese to be efficient. The Chinese, Dr. Jenkins found at first, were rather averse to operations of a major type. They had a very great dislike to the loss of limbs, and only consented when at last they discovered that lives were saved by it. It is also practically a new thing to get permission to open an abdomen. The foreign doctor found that some of his practice came to him because he had chloroform, or 'dreaming medicine' as they call it.

"This, then, was the pioneering work that Dr. Jenkins was engaged in before he went home for his first furlough in 1911. During his furlough he was as enthusiastic as ever in the interests of his beloved hospital. He made arrangements for installing an X-ray apparatus, and it is sad to think that the apparatus was well on its way out to him when he became ill. Other improvements were also thought over and arranged, the chief one being the building of a much-needed new hospital.

"When at home for a short time the news of the revolution came, and Dr. Jenkins volunteered to return to relieve the little band who were holding the fort. News came, however, that most of the missionaries were leaving for the coast, and so he remained at home for full furlough.

"On his return to Sianfu Dr. Jenkins found the work carried on by his colleague, Dr. Robertson, and immediately took up his share. In less than two months Dr. Robertson became ill with typhus fever. In addition to the whole hospital work that fell on his shoulders, Dr. Jenkins took his share in nursing the patient. Dr. Robertson had got the disease in the out-patient department, and subsequent events showed that Dr. Jenkins had also got the disease from the same source. At the end of four days Dr. Jenkins became ill, but he continued to work without letting anyone know. On the Friday, with a temperature of 103 deg., he saw about sixty out-patients. That night he went to bed with his Chinese servant sleeping in his room as the only attendant. Next day he was unable to get up. It was good to see how his Christian faith made him at once reconciled to God's will. He knew that his heart bore traces of former rheumatism; but if it was the call to other service he was ready to go. Only his ties to his wife and two little children made him hesitate for a moment, but he soon gained strength to leave them to the keeping of that same Jesus who was calling him.

"Two weeks passed and the fever left him. We thought he was going to be left to us here, but the strain upon his heart had been too much, and a week later he passed away. His wife and some of his fellow-missionaries were at his bedside. God was good in allowing Mrs. Jenkins, after a fortnight's journey, to spend the last three days with her husband.

"He was laid to rest in the Baptist Missionary Churchyard by his fellow-missionaries and many of the Chinese to whom he had brought the Gospel, and laid down his life in giving it. What a sad blank he has left in their lives and ours, whom he has left to perhaps reap some of what he has sown."

### Local Medical Notes.

